# Prognostic Factors for Prostate Cancer Endpoints Following Biochemical Failure: A Review of the Literature

**DOI:** 10.7759/cureus.238

**Published:** 2015-01-05

**Authors:** Tim Nguyen, R Gabriel Boldt, George Rodrigues

**Affiliations:** 1 Radiation Oncology, London Health Sciences Centre; 2 London Health Sciences Centre; 3 Department of Oncology, London Health Sciences Centre; Schulich School of Medicine & Dentistry, Western University, London, Ontario, CA

**Keywords:** prostate cancer, biochemical failure, biochemical recurrence, psa, clinical endpoints, clinical outcomes, prognostic factor, predictor, survival

## Abstract

Purpose: In the setting of biochemical failure (BCF) following primary treatment for prostate cancer, additional discrimination between clinically significant and non-clinically significant biochemical recurrence is critical in defining robust surrogate endpoints for prostate cancer and guiding salvage management decisions. We reviewed the literature to determine which prognostic factors are most significant for predicting prostate cancer-specific survival (PCSS), metastases-free survival (MFS), and/or overall survival (OS) after BCF.

Materials and Methods: A search of PubMed from 1980 to 2013 yielded 999 studies that examined prognostic factors predictive for PCSS, MFS, and/or OS in prostate cancer patients with BCF following primary treatment. Eligibility criteria for inclusion were: 1) examined a prostate cancer population in the setting of BCF without overt clinical relapse following primary treatment with radical prostatectomy or radiotherapy; 2) based analyses on patient parameters obtained prior to the initiation of salvage therapies; and 3) determined clinical prognostic factors that were significant prognostic measures for at least one of three clinically relevant endpoints: OS, PCS, or MFS.

Results: Nineteen eligible studies reported on 8,040 patients that experienced BCF from 1981-2013. The initial primary therapy was variable: radical prostatectomy alone (n=8), radiotherapy alone (n=4), radiotherapy/radical prostatectomy ± adjuvant therapy (n=5), and multiple treatment arms (n=2). There was also heterogeneity in which outcomes were assessed: PCSS (n=14), MFS (n=7), and OS (n=5). The prognostic factors most commonly found to be significant on multivariate analyses were PSA doubling time (PSADT), time to biochemical failure (TTBF), pathological Gleason score (pGS), and age.

Conclusions: Risk stratification in prostate cancer post-BCF is challenging because of limited predictive modeling that can determine which patients will optimally benefit from salvage therapy. Our systematic literature review has identified PSADT, TTBF, pGS, and age as the leading prognostic factors for the prediction of PCSS, MFS, and OS after BCF. We plan to leverage the Canadian ProCaRS database to perform predictive modeling using the putative findings in the present study in order to propose potential evidence-based surrogate endpoints for prostate cancer in the setting of BCF.

## Introduction

In 2014, an estimated 23,600 men will be diagnosed with prostate cancer in Canada, resulting in approximately 4,000 prostate cancer-specific deaths [1]. Of the men who are successfully treated with radical prostatectomy, about 35% will have a biochemical failure (BCF) [2]. Once BCF is established, the American Urological Association (AUA) has reported a median interval of five to 12 years until prostate cancer-specific mortality (PCM) [3].

The definition of BCF varies depending on the primary treatment. Following successful radical prostatectomy, the AUA defines BCF as two successive measurements of a serum PSA ≥ 0.2 ng/mL after undetectable PSA levels had been reached, which typically occurs at least six weeks postoperatively [3]. Applying a serum PSA cut-off of 0.4 ng/mL, however, was found to more closely correlate with disease progression [3]. In 2005, the American Society for Therapeutic Radiation and Oncology (ASTRO) Consensus Committee convened in Phoenix, Arizona, and proposed a definition for BCF following primary treatment with radiotherapy as an increase in PSA by ≥ 2 ng/mL above the treatment nadir [4]. Previously, the ASTRO definition for BCF was three consecutive rises after a treatment nadir had been established [5]. There is consequently a dichotomy amongst the definitions of BCF used which needs to be considered when comparing studies published before and after the 2005 amendment.

Although the natural history of recurrent prostate cancer is relatively slow, its contribution towards all-cause mortality (ACM) in the setting of clinical recurrence remains high, accounting for 77% of deaths in recurrences that occur within the first 15 years following treatment [6]. BCF is a common and important scenario in the management of prostate cancer. Deciding between management options at this clinical crossroads is challenging. Events of BCF are not all equal, with low-risk biochemical failure cohorts being identified as having a longer median time to BCF compared with higher risk groups (55 months vs 33 months) and improved five year overall survival as well [7]. This highlights the unreliability in using BCF alone as a surrogate endpoint for survival and the need for other clinical parameters to adequately risk-stratify patients. 

The objectives of this investigation were twofold. First, we sought to determine what prognostic factors have been identified in the literature as being significant or non-significant for PCM, distant metastasis (DM), and ACM. Second, we aimed to identify which prognostic factors were most consistently significant for the previously stated clinical outcomes that can then serve as the basis for developing future guidelines and risk stratification tools.

## Materials and methods

### Literature review

**Figure 1:**
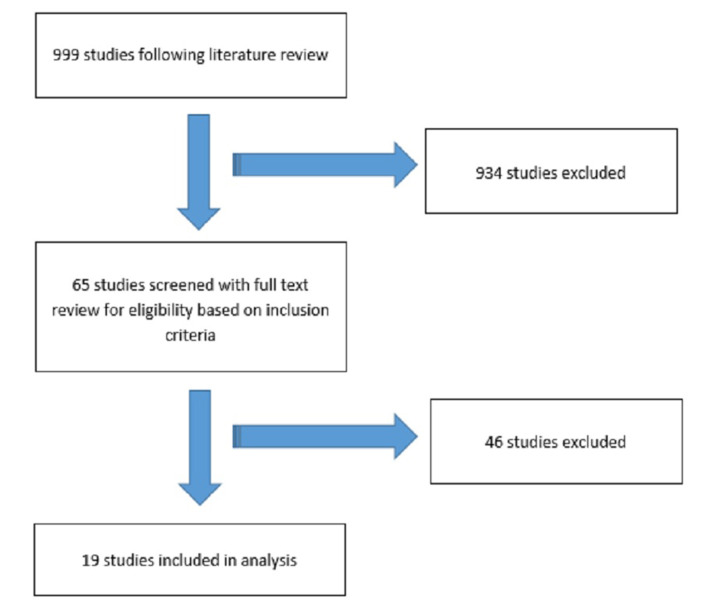
Search Strategy

We conducted a review of PUBMED from 1981-2013 with the following search strategy: (biochemical*[ti] OR PSA[ti] OR prostate specific antigen[ti]) AND (failure*[ti] OR recurrent*[ti] OR relapse*[ti]) AND prostatic neoplasms [mh]. Author TN screened the citations based on titles and abstracts to select relevant articles for full text review. Authors GBR and TN completed the full text screening according to inclusion and exclusion criteria. Disagreements were settled by discussion until 100% consensus was achieved. The literature review resulted in 999 studies for assessment. Initial screening of titles and abstracts yielded 65 studies. Following full article reviews, 19 studies fulfilled all eligibility criteria and were included in the present study for further analysis (Figure 1) [2, 6, 8-24].

### Inclusion criteria

We selected studies that fulfilled the following criteria:

1) Examined a prostate cancer population in the setting of BCF without overt clinical relapse following primary treatment with radical prostatectomy or radiotherapy;

2) Based analyses on patient parameters obtained prior to the initiation of salvage therapies;

3) Determined clinical prognostic factors that were significant prognostic measures for at least one of three clinically relevant endpoints: ACM, PCM, or DM.

Editorial letters, correspondences, and untranslated foreign reports were excluded. 

### Data abstraction

We abstracted the following data from all included studies: authors, year of publication, country of study origin, study design, definition of BCF, primary treatment, number of total patients, number of patients who experienced BCF, age, baseline PSA, baseline Gleason score (GS), baseline TNM staging, prognostic factors (abstracting data from both univariate and multivariate analyses), and survival outcomes (PCM, DM, ACM).

Informed patient consent was obtained at the time of treatment. No identifying patient data was used in this paper.

### Reporting outcomes

To illustrate the frequency at which potential prognostic factors were found to be significant or non-significant for a given outcome across included studies, an arbitrary, a priori scale was created (Table 1).

**Table 1:** Determining the frequency at which studies showed a significant relationship between a given prognostic factor and clinical outcome. *2:1 and 1:0 considered weakly significant, given the same difference in significant studies versus non-significant studies.

Degree of Significance Across Studies	Significance Ratio (SR)
Mostly non-significant	SR < 1 (Example: 1:2)
Weakly significant	1 < SR <2 * (Example: 3:2)
Moderately significant	SR = 2 (Example: 4:2)
Strongly significant	SR > 2 (Example: 3:1)
Indeterminate	SR = 1* (Example: 2:2)

For the purposes of this study, we coined the term *significance ratio (SR), *which represents the ratio between the number of studies showing a significant correlation between a given variable (e.g., PSADT) and a given clinical outcome (e.g., OS) and the number of studies demonstrating a non-significant finding for the same relationship. If the SR was less than 1 (e.g., 1:2), then the correlation between that variable and clinical outcome was considered *mostly* *non-significant. *If the SR for was equal to 1, then the relationship was regarded as indeterminate. With a SR greater than 1 but less than 2, the relationship was *weakly significant*. Finally, a SR equal to 2 was *moderately significant* and a SR greater than 2 was considered *strongly significant*.

An important note is that the described significance scale was not weighted. For example, a ratio of 2:1 (studies showing significance vs. studies not showing significance) is less convincing and more subject to chance than a ratio of 6:3. This should be considered when interpreting our results.

## Results

### Methodology

We identified 19 studies published between 1996 and 2012 that met all inclusion criteria, the majority of which were retrospective studies (84%). The remainder included two randomized control trials and one prospective study. Fourteen of the studies were conducted in the USA, two in Australia, one in Japan, and one in Canada. The key findings of each study are summarized in Table 2.

**Table 2:** Summary of Findings

Authors	Year	Primary Treatment	Definition BCF	Findings
Antonarakis, et al.	2011	RP	PSA ≥ 0.2ng/mL	On multivariate analysis, shorter PSADT was a significant predictor for ACM and DM. Increasing age at time of surgery was significant for ACM.
Antonarakis, et al.	2012	RP	PSA ≥ 0.2ng/mL	On multivariate analyses, higher GS (<6 vs. 7 vs. 8-10), and shorter PSADT (<3.0 vs. 3.0-8.9 vs. 9.0-14.9) were associated with increased risk of DM. PSADT was significant predictor of DM on multivariate analyses as a continuous variable as well.
Boorjian, et al.	2011	RP	PSA ≥4ng/mL	Increasing age at BCF, increasing GS, advanced tumor stage, and rapid PSADT were predictive for DM and PCM on multivariate analysis.
Boorjian, et al.	2012	RP + adjuvant radiotherapy	PSA ≥4ng/mL	Higher GS and shorter PSADT were significant predictors of systemic progression on multivariate analysis. PSADT < 6 months had an 11-fold increased risk of systemic progression vs. PSADT > 10 months
Buyyounouski, et al.	2008	EBRT	Nadir + 2ng/mL	Examined TTBF cut-off points of < 12 months, < 18 months, and < 24 months On multivariate analyses, for predicting DM only the < 18 mo cut-off was significant. For predicting PCM, all three cut-offs were significant.
Buyyounouski, et al.	2012	EBRT	Nadir + 2ng/mL	Shorter TTBF was predictive of ACM and PCM using 18 months as a cut-off. Greatest discriminatory power (using concordance indexes) was achieved with a model including TTBF, PSADT, and PSA nadir)
D’Amico, et al.	2003	RT	3 consecutive rises in PSA after nadir established.	On multivariate analyses, GS and pre-treatment PSA were significant predictors for PCM.
D’Amico, et al.	2006	RT ± complete androgen blockade	PSA of more than 1.0 ng/mL and increasing by more than 0.2 ng/mL on two consecutive measurements	Shorter PSADT (< 6mo, 6-12mo and >12mo) and younger age (< 75 years) at time of BCF were significant predictors of ACM and PCM
Denham, et al.	2008	EBRT alone vs EBRT + 3mo ADT vs EBRT + 6mo ADT	Nadir + 2ng/mL	TTBF and PSADT were significant predictors of PCM Best predictive power with TTBF cut-offs of < 1.5 and <2; PSADT cut-off of < 12 mo.
Denham, et al.	2009	EBRT alone vs EBRT + 3mo ADT vs EBRT + 6mo ADT	Nadir + 2ng/mL	Shorter PSADT and TTBF were strongest predictors for PCM. Older age at BCF was weakly significant with a low hazards ratio.
Freedland, et al.	2005	RP	PSA ≥ 0.2ng/mL	On multivariate analysis higher GS (≤ 7 vs ≥ 8-10), shorter TTBF (< 3 years), and shorter PSADT (< 3.0 vs 3.0-8.9 vs 9.0-14.9 vs ≥ 15.0) were the only significant predictors for PCM.
Freedland, et al.	2006	EBRT	PSA ≥ 0.2ng/mL	On multivariate analyses, shorter TTBF, shorter PSADT, and higher GS were significant predictors of PCM.
Freedland, et al.	2007	EBRT	PSA ≥ 0.2ng/mL	Shorter PSADT (< 3, 3-8.9, 9-14.9 and ≥15), earlier TTBF, and GS ≥ 8 were predictors for PCM and ACM. Older age at BCF was associated with ACM but not PCM.
Hachiya, et al.	2006	RP	Two consecutive detectable PSA levels ≥4 ng/mL	Earlier TTBF (cut-off two years) was associated with PCM and DM.
Kim-sing, et al.	2004	EBRT (37% received neoadjuvant/adjuvant HT for a median of 7.5 months)	2 consecutive rises above nadir measured minimal 1 month apart.	On multivariate analysis, only faster PSADT (< 3 mo, 3-6 mo, 6-12 mo and > 12 mo) and earlier intervention were significant for PCM.
Roberts, et al.	2001	RP	PSA ≥4ng/ml	On multivariate analysis, found only PSADT to be a significant predictor for local recurrence-free survival and DM.
Sandler, et al.	2000	EBRT	3 consecutive rises in PSA.	Statistical relationship was seen between PCM and two variables: 1) the slope of the ln PSA 2) relative PSA
Stock, et al.	2008	Brachytherapy	Nadir + 2ng/mL	On multivariate analyses, PSADT and TTBF were significant predictors for developing DM.
Wo, et al.	2009	EBRT alone vs. EBRT + ADT	two consecutive rises in PSA of > 0.2 ng/mL after nadir	Increasing PSA velocity at recurrence and moderate to high comorbidity were associated with increased risk of all-cause mortality.

There was heterogeneity in the definition of BCF between reports. There were three different definitions amongst studies that examined radical prostatectomy as the primary therapy. Five studies defined BCF as a single postoperative PSA value ≥ 0.2 ng/mL, three studies defined it as a single postoperative PSA value ≥ 0.4 ng/mL, and one study defined it as two consecutive PSA levels ≥ 0.4 ng/mL. The heterogeneity was even greater amongst studies where radiotherapy was the primary treatment. Five studies employed the ASTRO Phoenix definition of BCF, which is a rise in PSA level of ≥ 2 ng/ml above the nadir following primary treatment with radiotherapy +/- hormone therapy. Two studies abided by the former 1997 ASTRO definition as three consecutive rises in PSA following primary treatment with radiotherapy. The remaining three studies where radiotherapy was the primary treatment each employed different definitions, describing BCF as two consecutive rises of > 0.2 ng/mL above the nadir, PSA > 1.0 ng/mL and increasing by > 0.2 ng/mL on two consecutive measurements and three consecutive PSA rises, respectively. 

### Patient characteristics

The 19 eligible studies reported on 8,040 patients with BCF who received treatment between 1981 and 2010. Eight studies reported on patients whose primary therapy was radical prostatectomy alone. Four studies examined patients with external beam radiotherapy (EBRT) alone, five studies on EBRT with or without hormone therapy, and two studies were mixed treatment modality studies with multiple treatment arms. Eleven studies reported median age at BCF (64-75, overall median = 69), six studies reported mean age at BCF (59-72.5), and three studies did not report age at BCF.    

### Outcomes

Twelve studies examined a single clinical outcome, while seven studies reported on two or more outcomes. PCM was reported in 14 studies, DM was reported in seven studies, and ACM was reported in five studies. Nearly half the studies (9) reported the results of both univariate and multivariate analyses, five reported only univariate results, and five reported only multivariate results.

### Prognostic factors - Age

**Figure 2:**
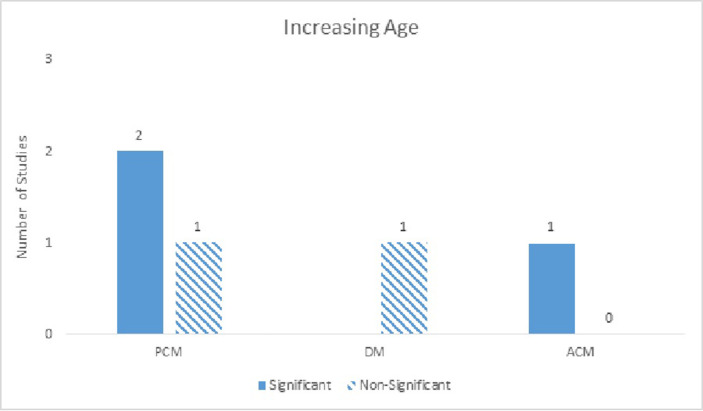
Increasing Age Number of studies showing age as a significant predictor for PCM, DM, and ACM on multivariate analyses. One study (not represented in the figure) found younger age to be associated with worse PCM and ACM

There were six studies that examined age as a prognostic indicator in patients following BCF, and there was fair amount of heterogeneity amongst the results. Five studies examined age at BCF, whereas one study examined age at the time of primary treatment [8]. Older age at BCF was weakly significant for ACM on both univariate and multivariate analyses with significance ratios of 1:0 for both. For PCM, older age at BCF was non-significant on univariate analysis (0:1) and weakly significant on multivariate analyses (2:1). Only one study examined the relationship between age and DM, and no significant correlation was found on multivariate analysis (0:1) (Figure 2). Antonarakis, et al. found a significant association between older age at the time of primary surgery and ACM [8]. In addition, one study found younger age was a significant predictor for worse PCM and ACM.

### Prognostic factors - TNM staging

**Figure 3:**
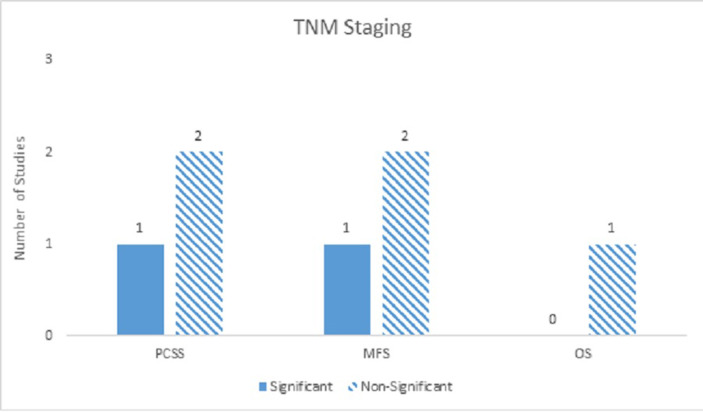
TNM Staging Number of studies showing TNM staging as a significant predictor for PCM, DM, and ACM on multivariate analyses.

Seven studies included TNM staging in their analyses. For PCM, TNM staging was indeterminate on univariate analyses with a significance ratio of 1:1 and mostly non-significant on multivariate analyses with a significance ratio of 1:2. With regards to DM, univariate analysis was weakly significant (1:0), while multivariate analyses were most non-significant (1:2). Finally, with OS, univariate analysis was weakly significant (1:0) and multivariate analysis was mostly non-significant (0:1) (Figure 3).

### Prognostic factors - Gleason score

**Figure 4:**
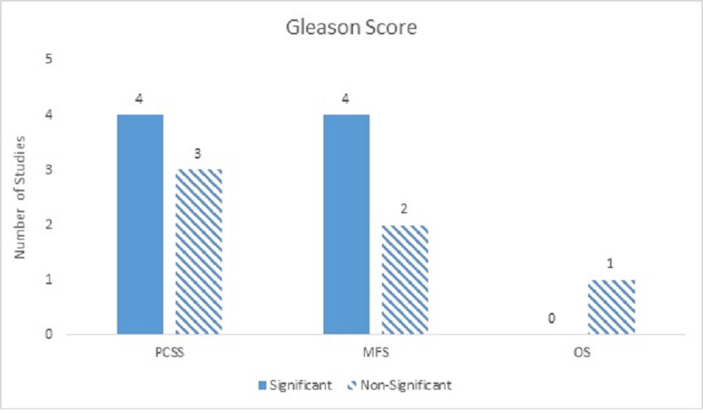
Gleason Score Number of studies showing GS as a significant predictor for PCM, DM, and ACM on multivariate analyses.

Thirteen studies examined GS as a prognostic factor. It was found to be widely significant across studies with univariate analyses across all clinical outcomes; however, there was more heterogeneity and less convincingly significant results with multivariate analyses. On univariate analyses, GS was a moderately significant predictor for PCM and ACM with significant ratios of 3:0 and 2:0, respectively. DM was strongly significant on univariate analyses (5:0). On multivariate analyses, GS was a strongly significant predictor for DM (4:2), moderately significant for PCM (4:3), and mostly non-significant for ACM (0:1) (Figure 4).

### Prognostic factors - PSA doubling time

**Figure 5:**
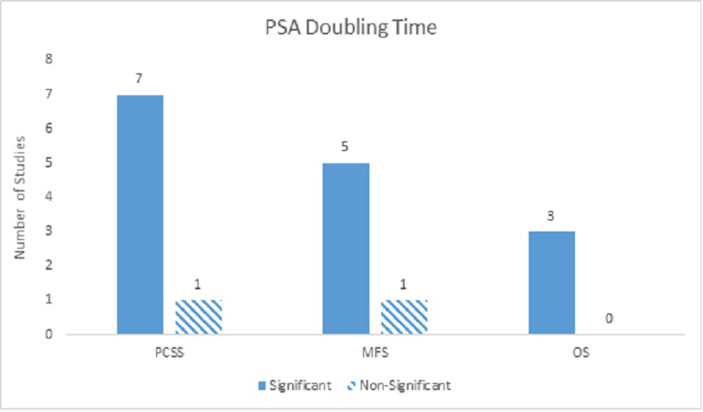
PSA Doubling Time Number of studies showing PSADT as a significant predictor for PCM, DM, and ACM on multivariate analyses.

Fifteen studies included PSADT in their analyses and demonstrated this parameter to be strongly significant in nearly all clinical outcomes in both univariate and multivariate analyses. For PCM, PSADT was strongly significant on univariate analyses, with a significance ratio of 4:1, and multivariate analyses with a significance ratio of 7:1. PSADT was also strongly significant for DM on both univariate analyses with a significance ratio of 5:0 and multivariate analyses with a significance ratio of 5:1. With regards to ACM, PSADT was strongly significant on univariate analyses (4:0), but only moderately significant on multivariate analyses (3:0) (Figure 5).

### Prognostic factors - Time to biochemical failure

**Figure 6:**
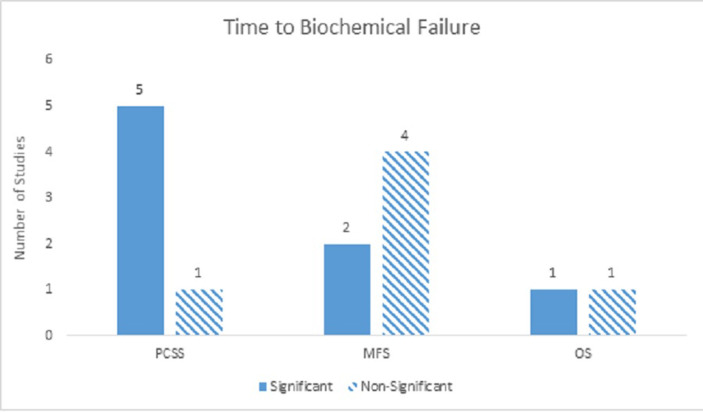
Time to Biochemical Failure Number of studies showing TTBF as a significant predictor for PCM, DM, and ACM on multivariate analyses.

Fourteen studies examined TTBF. For PCS, TTBF was a strongly significant prognostic factor, with a significance ratio of 4:1 on univariate analyses and 5:1 on multivariate analyses. TTBF was also a strongly significant predictor for DM, but only on univariate analysis with a significance ratio of 5:1. On multivariate analysis, the relationship between TTBF and DM was mostly non-significant with a significance ratio of 2:4. For OS, TTBF was a moderately significant predictor on univariate analyses (3:1) and indeterminate on multivariate analyses (1:1) (Figure 6).

Using recursive-partitioning analysis, Buyyounouski, et al. demonstrated TTBF was significant as a categorical value as well as a continuous value [13]. Of the factors they examined, TTBF was found to be the best predictor for DM, and interestingly, PSADT was not significant. On the other hand, Denham, et al. showed that both PSADT and TTBF were the most important factors for predicting PCS [17]. 

### Prognostic factors - Initial PSA

**Figure 7:**
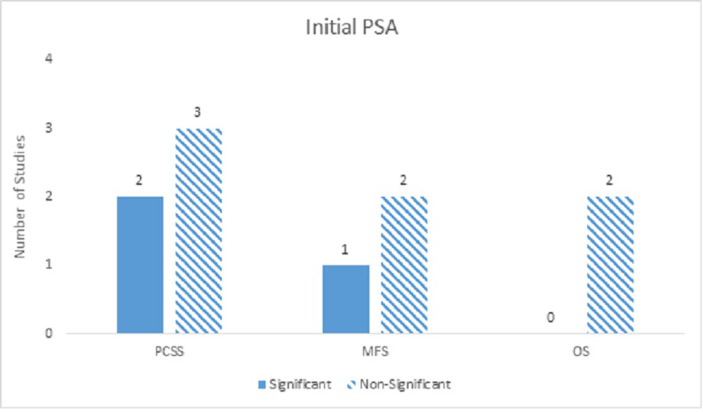
Initial PSA Number of studies showing iPSA as a significant predictor for PCM, DM, and ACM on multivariate analyses.

Eight studies looked at the pre-treatment iPSA, which was found to be mostly non-significant across all clinical outcomes in both univariate and multivariate analyses. The exception was PCS on univariate analysis, which was indeterminate (Figure 7).

### Prognostic factors - Other

Buyyounouski, et al. looked at PSA nadir as a prognostic indicator and found a significant relationship with DM on multivariate analysis when TTBF was included as a categorical variable, but not as a continuous variable [12].

Antonarakis, et al. examined factors predictive of DM in patients with BCF post-radical prostatectomy. They demonstrated that race, lympho-vascular invasion, and seminal vesicle invasion were significant on univariate analysis but not on multivariate analysis [9].

## Discussion

The primary objective in the current study was to determine which clinical variables were most promising as prognostic indicators for prostate cancer outcomes, specifically at the time of BCF. Accordingly, we were interested in the variables that scored favorably on our significance scale: either moderately significant or strongly significant. On univariate analysis, only Gleason score (DM), PSADT (PCM, DM, and ACM) and TTBF (PCM and DM) were identified as strongly significant for clinical outcomes. Moderate significance was observed for GS (PCM and ACM) and TTBF (ACM). On multivariate analyses, GS (DM), PSADT (PCM and DM), and TTBF (PCM) were again the only strongly significant factors, with moderate significance noted for PSADT (ACM). Based on the literature at the time of our review, we have identified GS, PSADT, and TTBF as the clinical parameters with the most potential as surrogate endpoints for prostate cancer outcomes. Of all these factors, PSADT was the most consistent, demonstrating strong significance across nearly all clinical outcomes in both univariate and multivariate analyses. When evaluating a patient who has developed biochemical failure without evidence of clinical progression, we recommend examining these potential surrogate endpoints and considering salvage therapy in patients with higher risk. In particular, high risk features that have been shown to be significantly associated with worse clinical outcomes include a GS ≥ 8, PSADT < 3-6 months, and TTBF < 1.5-3 years. Additional research is required to determine more definitive evidence-based guidelines.

A particularly relevant study from Memorial Sloan Kettering provides support for our findings but was unfortunately published after the timeframe set for our original search strategy. Zumsteg, et al. conducted a retrospective single institution study of men who developed BCF after definitive EBRT. Similar to our findings, on multivariate analysis, they found a GS ≥ 8, clinical T stage 3b-4, faster PSADT, and shorter TTBF were significant independent predictors of prostate cancer outcomes. Specifically, GS, T stage, and PSADT were predictors for DM, PCM, and ACM. TTBF was only a significant predictor for DM. Also in accordance with our review, Zumsteg, et al. determined optimal cut-off values for PSADT and TTBF as 3.2 months and 2.9 years, respectively [25].

Although we identified PSADT as the most consistently significant prognostic factor for clinical outcomes in the setting of BCF, its use in assessing the need for salvage therapy is not without limitations. In order to calculate PSADT, multiple PSA measurements are required, potentially necessitating relatively long follow-up intervals before a complete assessment can be established. Initiating salvage therapy will affect PSA levels leading to inaccurate PSADT calculations; however, not surprisingly, there is a reluctance to postpone second-line therapy for this reason alone. Hamilton, et al. studied 535 patients and found 35% did not have a calculable PSADT either due to missed follow-up appointments or the imitation of salvage therapy before PSADT calculations were completed. In addition, they reported that patients with a calculable PSADT tended to fall within a lower-risk cohort with longer time to biochemical failure, lower BMI, and more favorable pathologic features, identifying the possible risk of selection bias [26]. As a counterpoint, several of the studies reviewed identified PSADTs of three months or six months as thresholds for higher risk groups most susceptible to adverse outcomes. Measurements can arguably be obtained within these timeframes without unreasonable delay to at least determine if patients fall within the high-risk group, which can aid treatment decisions.

In the majority of the studies reviewed, patients eventually received salvage therapy as per the treating physician’s clinical judgment. Consequently, this heterogeneity across studies may have an impact on eventual clinical outcomes, which is an important limitation to our study. In addition, most studies were retrospective in design and thus carry the potential for patient and treatment selection biases.  

## Conclusions

Risk stratification of prostate cancer patients in the setting of BCF is challenging because of limited predictive modeling that can determine which patients will optimally benefit from salvage therapy. We have identified Gleason score, TTBF, and PSADT as the most consistently significant prognostic factors for PCM, DM, and ACM in the literature. These findings will guide current efforts to perform predictive modeling using the ProCaRS database and propose evidence-based surrogate endpoints and management guidelines for prostate cancer in the setting of BCF.
